# Effect of Surgical Technique on the Microstructure and Microcirculation of the Small Intestine Stump during Delayed Anastomosis: Multimodal OCT Data

**DOI:** 10.17691/stm2021.13.4.04

**Published:** 2021-08-28

**Authors:** E.B. Kiseleva, M.G. Ryabkov, M.A. Sizov, E.L. Bederina, A.D. Komarova, A.A. Moiseev, M.V. Bagryantsev, A.N. Vorobiev, N.D. Gladkova

**Affiliations:** Senior Researcher, Scientific Laboratory of Optical Coherence Tomography, Research Institute of Experimental Oncology and Biomedical Technologies; Privolzhsky Research Medical University, 10/1 Minin and Pozharsky Square, Nizhny Novgorod, 603005, Russia; Associate Professor, Leading Researcher, University Clinic; Privolzhsky Research Medical University, 10/1 Minin and Pozharsky Square, Nizhny Novgorod, 603005, Russia; Surgeon; City Clinical Hospital No.30, 85A Berezovskaya St., Nizhny Novgorod, 603157, Russia; Pathologist, Junior Researcher, University Clinic; Privolzhsky Research Medical University, 10/1 Minin and Pozharsky Square, Nizhny Novgorod, 603005, Russia; Student, Department of Biophysics; National Research Lobachevsky State University of Nizhni Novgorod, 23 Prospekt Gagarina, Nizhny Novgorod, 603950, Russia; Laboratory Assistant, Laboratory of Fluorescent Bioimaging, Research Institute of Experimental Oncology and Biomedical Technologies; Privolzhsky Research Medical University, 10/1 Minin and Pozharsky Square, Nizhny Novgorod, 603005, Russia; Senior Researcher, Laboratory of Highly Sensitive Optical Measurements; Federal Research Center Institute of Applied Physics of the Russian Academy of Sciences, 46 Ulyanova St., Nizhny Novgorod, 603950, Russia; Surgeon; City Clinical Hospital No.30, 85A Berezovskaya St., Nizhny Novgorod, 603157, Russia; Surgeon; City Clinical Hospital No.30, 85A Berezovskaya St., Nizhny Novgorod, 603157, Russia; Professor, Head of the Scientific Laboratory of Optical Coherence Tomography, Research Institute of Experimental Oncology and Biomedical Technologies; Privolzhsky Research Medical University, 10/1 Minin and Pozharsky Square, Nizhny Novgorod, 603005, Russia

**Keywords:** acute intestinal ischemia, multimodal optical coherence tomography, MM OCT, cross-polarization optical coherence tomography, CP OCT, optical coherence angiography, OCA, intestinal viability, enterostomy, enteroanastomosis

## Abstract

**The aim of the study** was to use multimodal optical coherence tomography (MM OCT) to evaluate microstructure and microcirculation in the proximal and distal sections of the intestine relative to the resected area in acute mesenteric ischemia.

**Materials and Methods:**

The study was carried out using three groups of male Wistar rats weighing 270–435 g (n=18). Acute occlusive arterial ischemia of the small intestine was initiated in all animals. After 80–90 min, the ischemic non-viable part of the intestine was resected, and the operation was completed by stoma exteriorization (group 1, n=6), by applying purse-string sutures (group 2, obstructive resection, n=6), or by internal drainage of the proximal and distal ends of the small intestine (group 3, bypass, n=6). Relaparotomy and anastomosis formation were performed 2 days later.

With the help of MM OCT at each stage of the surgical intervention, images were obtained from the serous membrane side: the intestinal wall microstructure (layers) was viewed using cross-polarization OCT (CP OCT) and the intramural circulation — using optical coherent angiography (OCA). The MM OCT images obtained from the terminal intestine sections immediately after resection and 2 days later (before the anastomosis formation) were compared between the experimental groups, as well as with the pre-ischemic data (norm). All resected sections of the intestine were then histologically examined. The MM OCT data were compared with the histological and intravital macroscopy data.

**Results:**

As a result of studying the intestinal wall microstructure by *in vivo* CP OCT, it was found that during ostomy (group 1) and obstructive resection (group 2), the images showed signs of tissue edema and destructive changes in the mucous membrane that were confirmed histologically, while with bypass surgery (group 3), there were minimal changes as compared with the norm.

According to the OCA data, on day 2 of ostomy in the proximal and distal segments of the intestine, there was a noticeable disappearance of small and medium blood vessels; mainly large arteries and veins could be visualized. Following obstructive resection (purse-string suturing) or bypass surgery, the most noticeable changes (a decrease in the number of visualized blood vessels) were observed in the distal part of the intestine. The L index calculated from OCA images and characterizing the total length of the intramural perfused vasculature, showed a statistically significant decrease during ostomy: 12.18 [10.40; 14.20] μm — in the proximal and 10.67 [7.98; 13.05] μm — in the distal section; for comparison, the L index before ischemia was 18.90 [17.98; 19.73] μm and 18.74 [17.46; 19.90] μm, respectively (p=0.0001). In obstructive resection (group 2), statistically significant differences in the L parameter were found only for the distal bowel section: 16.39 [12.37; 18.10] μm compared with 18.74 [17.46; 19.90] μm before ischemia (p=0.041). After bypass surgery (group 3), there were no significant deviations in the L index.

**Conclusion:**

By using MM OCT, we found that in treating the remaining sections of the intestine after its emergency resection for acute mesenteric ischemia, the type of surgical technique determines the tissue structure in the period before the delayed anastomosis is applied.

The least pronounced and most balanced changes occur in the proximal and distal segments of the intestine when operated using the bypass technique. However, to recommend this type of surgery, the development of reliable, safe, and effective bypass instruments is needed.

## Introduction

Emergency resection of ischemic bowel remains the most common surgical operation in patients with acute mesenteric ischemia (AMI), and its frequency has increased significantly over the past 25 years [1]. According to the generally accepted protocol, the primary operation is not completed by imposing an enteroanastomosis; therefore, the post-surgery period is associated with a more or less prolonged disruption of the intestinal continuity [2, 3]. Depending on a specific clinical situation, the interrupted intestinal passage can last from several days (after obstructive bowel resection and a delayed anastomosis) to several months or years (with end enterostomy) [2]. Questions about how to treat the stump of the resected bowel in the interoperative period before the delayed anastomosis is applied, remain a subject of discussion. One of the important stages in this discussion was the release in 2018 of the Russian National Clinical Guidelines “Acute Vascular Bowel Diseases in Adults” [3], based on the international guidelines for the treatment of acute intestinal ischemia [2, 4]. They recommend abandoning the traditional enterostomy and choosing obstructive bowel resection followed by temporarily putting the closed stumps into the abdominal cavity until a second operation is performed and an anastomosis is applied. One of the most important reasons for avoiding enterostomy after AMI is the risk of complications associated with the stoma existence even for a short period. Among the most dangerous early complications of enterostomy are ischemia or necrosis of the intestine, peristomal purulent-necrotic lesion of the abdominal wall, and electrolyte-fluid imbalance [5–7]. Obstructive bowel resection followed by placing the stumps into the abdominal cavity may reduce or eliminate these risks; however, but it may lead to accumulation of chyme in the proximal section of the blocked intestine and failure of getting it to the distal part.

At the same time, the earliest possible restoration of the intestinal passage after resection is a key factor for decreasing the mortality and reducing the rehabilitation period [8, 9]. The early restoration of intestinal passage is more advantageous than intestinal ostomy. In the case of active nutritional support to a patient with a stoma, parenteral nutrition increases the risk of cholestasis and liver failure; only restoration of the enteral digestion leads to restoration of liver function [10]. In patients with an ultrashort small intestine remaining after emergency resection, restoration of the intestinal passage by imposing an anastomosis between the jejunum and the colon reduces the risk of recurrent ischemic damage and leads to the cancellation of parenteral nutrition in 35–50% of cases within a year [11]. However, the rapid restoration of intestinal passage by applying a primary anastomosis after emergency resection of ischemic intestine is unacceptable for most patients with AMI. In these patients, the risks and complications of an anastomotic leak are greater than the potential benefits of early restoration of the digestive function [2].

The issue of optimal surgical strategy early after AMI has not been fully resolved. There are three major approaches to the treatment for urgently resected ischemic bowel: 1) stoma exteriorization; 2) obstructive bowel resection with dropping the stumps into the abdominal cavity; 3) early restoration of the bowel passage throughout the entire intestine. To reduce the number of post-resection complications and incompetent anastomoses, it is important to know how each of these three techniques affects the microcirculation and microstructure of the intestinal wall.

Among the methods that allow intraoperative visualization of microcirculation, the most common one is fluorescence imaging with the indocyanine green as a fluorophore [12–14]. Tissue perfusion can be assessed using laser Doppler flowmetry [15, 16], laser speckle contrast imaging (LSCI) [17, 18], sidestream dark field (SDF) microscopy [19, 20], incident dark field (IDF) microscopy [21], and hyperspectral imaging (HSI) [22]. However, all the above methods are capable of assessing only the microcirculatory component of the intestinal wall (and in most cases, from the organ surface), allowing the measurement of tissue oxygenation or blood flow, but they do not provide information on structural tissue damage/necrosis [14]. The inability to directly visualize morphological changes in ischemic tissues is a significant drawback of the methods mentioned above. Circulatory disorders in acute intestinal ischemia encompass occlusive vascular obstruction, non-occlusive hypoperfusion, hyperdilation, and a spasm of intramural vessels in the ischemic zone, as well as compensatory collateral blood flow [23, 24]. Therefore, the data describing only one of the blood circulation parameters (volumetric blood flow rate, density of the vascular bed, etc.), without information on tissue microstructure and localization of functioning blood vessels may be insufficient. Besides, ischemic changes do not occur synchronously in the intestinal wall layers: structural damage in the deep layers of the wall can precede the complete cessation of blood circulation in the surface layers [25]. This situation can further complicate the interpretation of momentary microcirculation indices; therefore, the task of simultaneous monitoring of blood circulation and tissue structure becomes even more important.

This task can be accomplished by using multimodal optical coherence tomography (MM OCT) [26, 27]. The method is commonly applied to various fields of medicine [28–30]. Our group was the first to use MM OCT to study the intestinal wall in AMI with trans-serous access in animals [31, 32] and in the clinic [33, 34]. The results make us believe that MM OCT objectifies the observed changes in the small intestine under AMI and can be recommended for diagnosing microcirculation disorders (visualization of functioning blood vessels) and structural organization (visualization of edema and necrosis) of the intestinal wall tissues when determining the boundaries between non-viable and viable areas.

**The aim of the study** was to use multimodal optical coherence tomography to evaluate microstructure and microcirculation in the proximal and distal sections of the intestine after its emergency resection following acute mesenteric ischemia.

## Materials and Methods

The study was carried out with 18 male Wistar rats weighing 270–435 g in full compliance with the ethical principles established by the European Convention for the Protection of Vertebrate Animals used for Experimental and Other Scientific Purposes (Strasbourg, 2006); the protocol was approved by the Ethics Committee of the Privolzhsky Research Medical University (Nizhny Novgorod, Russia). All procedures were performed under general anesthesia induced with a mixture of 3.5% Zoletil and 2% xylazine hydrochloride, injected intramuscularly. At the beginning of the experiment, the animals were divided into three groups (n=6 in each) according to the three ways of treatment for the resected intestine. Thus, in group 1, the proximal section of the intestine was connected to a stoma and the distal section was blocked; in group 2, obstructive bowel resection was performed with the proximal and distal bowel sections blocked and placed into the abdominal cavity; in group 3, bowel bypass surgery was performed.

**Figure 1:**
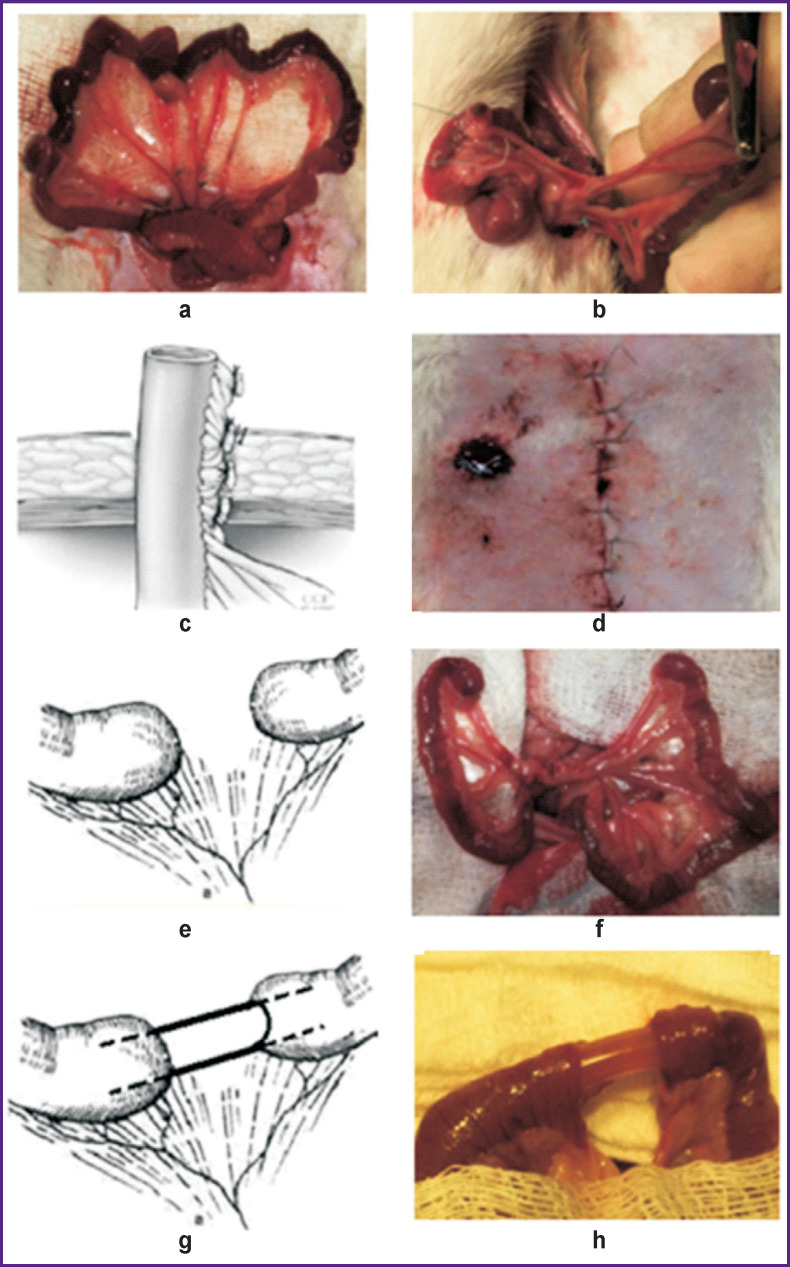
The initial stages of experimentation on the effect of surgical technique on microstructure and microcirculation of the small intestine stump in the delayed anastomosis surgery: (a) AMI modeling; (b) intestine tumps after resection; (c), (d) group 1, intestine tumps ostomy, scheme (c) and photo (d); (e), (f) group 2, obstructive bowel resection, scheme (e) and photo (f); (g), (h) group 3, bowel bypass, scheme (g) and photo (h)

At the first stage of the experiment, the animals in all three groups underwent a midline laparotomy; then, a loop of the jejunum, localized 20 cm distal to the duodenojejunal junction was pulled up into the wound. To simulate AMI, the animals were subjected to full segmental turnstile ischemia of the small intestine (tour-vessel occlusion model in the rat [35]): the branches of a. mesenterica cranialis were isolated and ligated thus interrupting the blood supply to a 7–10 cm long section of the intestine (Figure 1 (a)). The cessation of blood flow through the arteries was monitored visually — by vessel emptying and cessation of vascular pulsation distal to the ligation point. In addition, optical coherent angiography (OCA) was used to reveal a sharp decrease in the number of intramural blood vessels. This condition of acute bowel ischemia was maintained for 80–90 min, after which the ischemic portion of the bowel was resected (Figure 1 (b)). Then, one of the three types of treatment for the remaining intestine was tested (Figure 1 (c–h)).

In group 1 (ostomy), the proximal section of the intestine was brought out to the anterior abdominal wall to form an end enterostoma; the distal section was closed with a purse-string suture and placed into the abdominal cavity. In group 2 (obstructive resection), the proximal and distal sections of the intestine were closed with a purse-string suture and placed into the abdominal cavity. In group 3 (bypass), a shunt made of a PVC tube with a diameter of 5 mm and a length of 50 mm was installed between the proximal and distal sections of the intestine. The shunt was attached to the stumps with a purse-string suture. After bypass surgery, the intestinal stump was placed into the abdominal cavity. In all groups, at the final stage, the laparotomy wound was sutured in layers. In the postoperative period, the animals were not fed; to replenish fluid loss, 50 ml of saline per day was injected subcutaneously.

Two days after completing the first stage, the second stage was initiated: under anesthesia, relaparotomy was performed, the proximal and distal bowel sections were pulled up into the surgical wound, and then assessed using *in vivo* MM OCT. This method allows for real-time observation of tissue microstructure (cross-polarization mode — CP OCT) and its microcirculation (angiography mode — OCA). After that, the intestine was resected at a distance of 1 cm from the edge and taken for histological examination; the removed section was replaced with an inter-intestinal anastomosis.

MM OCT studies on the resection border tissues were also carried out at the first stage of the experiment before and after simulating AMI, to confirm the viability of the remaining tissues.

The MM OCT method and the research technique were described in detail in our earlier works [31, 33, 36, 37]. A high-speed spectral multimodal optical coherence tomograph (Federal Research Center Institute of Applied Physics of the Russian Academy of Sciences, Nizhny Novgorod, Russia) operating at a wavelength of 1310 nm with a spectral width of 100 μm and a power of 2 mW was used [36, 37]. The longitudinal resolution was 10 μm, the depth resolution — 15 μm, the scanning depth in air — ~1.7 mm, and the scanning speed was 20,000 A-scans per second. The MM OCT device is equipped with a flexible fiber optic probe, which ends with removable lens (outer diameter is 8 mm). The intestinal tissue was scanned using a contact method; the recording of one data volume (2.4×2.4×1.3 mm) took 26 s. Two types of images were obtained: 1) 3D structural images, from which any of 512 cross-sectional (transverse view) or *en face* (top view) scans could be selected and saved in co- or cross-polarization modes; 2) angiographic (OCA) images — a two-dimensional *en face* (top view) picture of the vascular network that could be contrasted from the background by using a high-frequency filter [37]. In this case, areas of blood flow (movement of erythrocytes) were visualized. Areas without blood flow are not visualized. The smallest vessel diameter that can be seen is 15 μm.

At the stage of processing, OCA images were visually and quantitatively assessed. The quantitative assessment was based on calculating the total length of blood vessels (L (μm)). To this end, the obtained two-dimensional OCA images were binarized followed by automatic sketching of the vessel skeleton and counting the number of pixels that made up this skeleton. The described transformations were carried out using the original program (Federal Research Institute of Applied Physics of the Russian Academy of Sciences), written in the Anaconda 4.3.1 mathematical environment (Python v. 3.6).

Structural (two-dimensional CP OCT and three-dimensional OCT) images were assessed visually: changes in the layering and thickness of the intestinal wall were assessed just after the AMI simulation and 2 days after surgical treatment of the intestinal stump; the results were compared to those obtained before ischemia.

For all areas of the small intestine studied using MM OCT, a histological assessment was then performed. Histological sections of the intestine were stained with hematoxylin and eosin and morphologically examined (Nikon Eclipse Ci microscope, DS-Fi 2 camera; Nikon, Japan).

**Statistical data processing** was performed using the IBM SPSS Statistics 20 software package. The normal distribution of the quantitative data was checked using the Kolmogorov–Smirnov test. None of the value sets obeyed the normal distribution pattern. Therefore, the assessment of statistical significance of differences was performed using nonparametric methods. To compare the indicators, the Kruskal–Wallis test was used. The parameters given below have the following designations: Me is the median, Q1 is the upper quartile, Q3 is the lower quartile, the min and max are the minimum and maximum values of the variable, n is the volume of the analyzed subgroup, p is the value of the statistical significance of differences. The critical value of the significance level was 5% (p≤0.05). When using multiple comparisons, the level of significance of differences is indicated as the adjusted value (p_adjusted_=p·m; where p is obtained from the comparison analysis, and m is the number of comparisons).

## Results

### Macroscopic changes in the intestinal wall resulting from different surgical techniques

**Figure 2:**
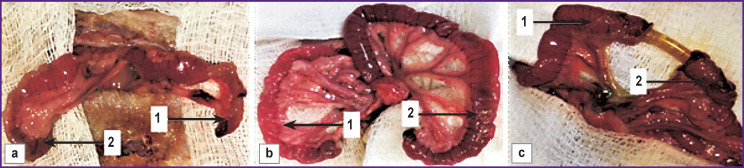
Macroscopic picture of the intestinal wall 2 days after resection: (a) group 1, the proximal and distal sections of the small intestine after ostomy are brought out into the laparotomy wound; (b) group 2, the proximal and distal sections of the intestine, closed and placed into the abdominal cavity for 2 days, after which brought out into the laparotomy wound; (c) group 3, the shunted ends of the intestine were brought out into the laparotomy wound; (*1*) proximal end of the intestine; (*2*) distal end of the intestine

Visual comparison between the proximal and distal bowel sections showed significant differences in their microstructures. In group 1, the proximal part (connected to the stoma) was a collapsed, pale, edematous piece of intestine with areas of hemorrhage. The distal portion had a similar diameter, and a less active peristalsis (Figure 2 (a)).

In group 2, the differences between the proximal and distal bowel portions were much more pronounced. The blocked proximal segment was found markedly dilated in all animals of this group; it was moderately tense, the wall looked hyperemic and edematous. On the other hand, the distal portion was collapsed and pale; its wall was thinned (Figure 2 (b)). After bowel bypass surgery, no histological differences between the proximal and distal portions were found.

In all animals of group 3, the bowel sections had the same diameter, color, and peristaltic activity (Figure 2 (c)).

### Microstructure of the intestinal wall in vivo: comparison between CP OCT and histological data

**Figure 3:**
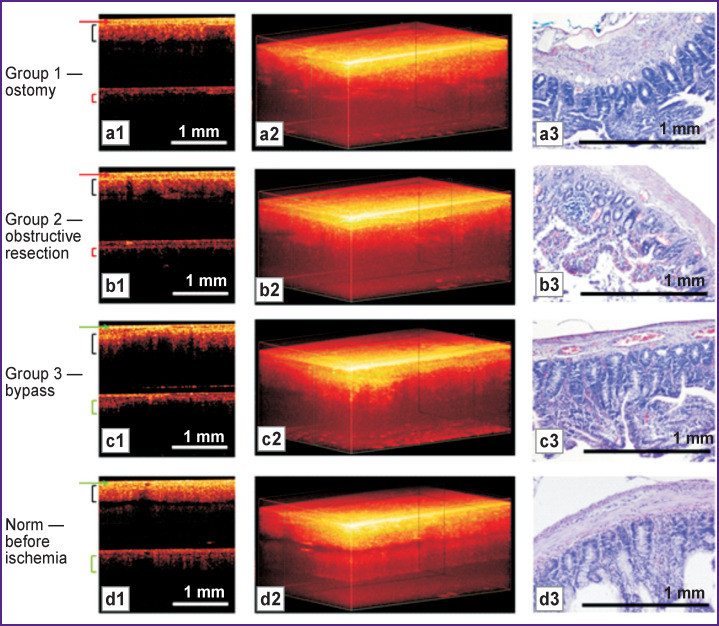
Microstructure of the intestinal wall 2 days after resection (the proximal portion of the intestine is shown): (a1), (b1), (c1), (d1) cross-sectional CP OCT images: upper part — image in co-polarization, lower part — image in cross-polarization; (a2), (b2), (c2), (d2) 3D OCT images; (a3), (b3), (c3), (d3) corresponding histology, hematoxylin and eosin staining. Green arrows indicate the non-thickened serous membrane: normal (d1) and after bowel bypass (c1); red arrows indicate the serous membrane thickened due to tissue edema during ostomy (a1) and obstructive resection (b1). Brackets for (a1), (b1), (c1), (d1) denote the mucosal layer of the intestinal wall. Cross-polarized images show a significant decrease in the signal from the mucous membrane under ostomy (a1) and obstructive resection (b1) groups compared with the normal (d1) and bowel bypass (c1) groups

According to the CP OCT data, in ostomy (group 1) and obstructive resection (group 2), a noticeable thickening of the first (serous) layer occurred (Figure 3 (a1), (b1)); in addition, the mucus layer was disrupted (the villous boundary line became rough), as clearly appeared in 3D images (Figure 3 (a2), (b2)). In the cross-polarization image, a significant decrease in the depth of tissue visualization after AMI (see Figure 3 (a1, b1)) compared to the norm (Figure 3 (d1)) was observed. These changes in tissue structure corroborated with a histologically detected edema of the intestinal wall, with partial detachment and desquamation of the mesothelium, as well as partial destruction and necrosis of the villi (Figure 3 (a3), (b3)).

After bowel bypass surgery (group 3), minimal histological changes were observed in all the membranes of the small intestine (Figure 3 (c3)). Likewise, the structure of CP OCT images was closely similar to the norm: the villi characteristic pattern could be clearly seen on the 3D images (Figure 3 (c2)), and the cross-section images; signs of edema were minimal (Figure 3 (c1)).

### Microcirculation in the proximal and distal portions of the intestine 2 days after surgery, according to OCA data

**Figure 4:**
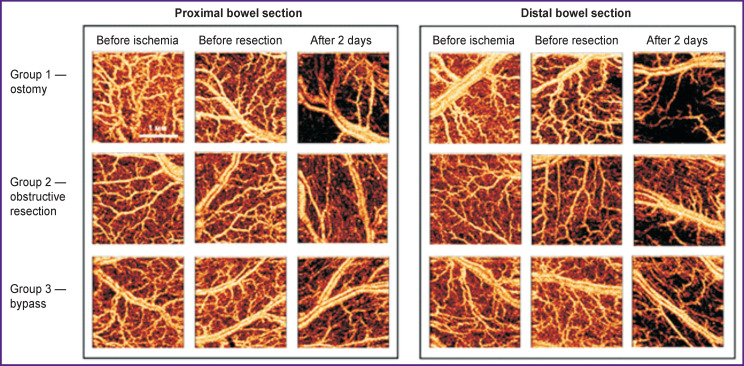
Microcirculation in the intestinal wall of the proximal and distal bowel sections distant from the resection zone after acute intestinal ischemia: OCA images obtained before ischemia, before resection, and after 2 days, depending on the method of treatment of the intestinal stump

**Figure 5:**
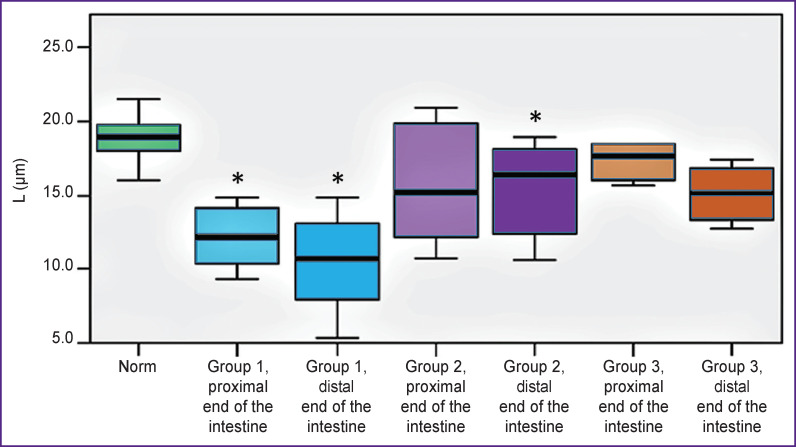
The total length of perfused intramural blood vessels (L index) calculated from OCA images of the small intestine under different treatments of the stump * Statistically significant differences between the groups and the norm (Kruskal–Wallis test)

The state of microcirculation in the intestinal wall in the proximal and distal sites remained after the resection were assessed visually (Figure 4) and quantitatively (Figure 5).

Before bowel resection, a barely visible change in intramural microcirculation (loss of few blood vessels) following the ischemic episode was observed (see Figure 4); that indicated a normal blood supply to the post-ischemic zones. On day 2 after ostomy, a noticeable loss of medium and small size vessels occurred in both the proximal and distal sections, where mainly large arteries and veins remained visible (see Figure 4, first row of images). After obstructive resection (with purse-string suturing) and after shunting, the most noticeable changes (a decrease in the number of visualized blood vessels) were observed in the distal end of the bowel (see Figure 4, second and third rows of images).

The L index characterizing the total length of the perfused intramural vasculature differed between the groups (see Figure 5). Immediately after excision of the ischemic section of the intestine, the total length of blood vessels in the proximal and distal remnants insignificantly decreased in all groups. In the proximal portion of the intestine, the L index values decreased from 18.90 [17.98; 19.73] to 18.49 [16.80; 19.82] μm (p_adjusted_=0.876); in the distal part — from 18.74 [17.46; 19.90] to 16.05 [12.56; 19.39] μm (p_adjusted_=0.254).

Two days after bowel ostomy (group 1), the L index significantly (p_adjusted_=0.0001) decreased compared to the initial level in both the proximal and distal bowel sections and reached 12.18 [10.40; 14.20] and 10.67 [7.98; 13.05] μm, respectively. In group 2, where both stumps were closed and placed in the abdominal cavity, the perfusion index L in the distal section of the intestine significantly decreased to 16.39 [12.37; 18.10] μm the proximal or distal parts of the intestine: the values (p_adjusted_=0.041). After bypass surgery (group 3), there were 17.69 [16.08; 18.43] and 15.11 [13.28; 16.85] μm, were no significant changes in the L index in either respectively (see the Table).

**Table:** Changes in the L index in the studied groups

Group	The of proximal the intestine part	The of the distal intestine part	Kruskal–Wallis test, p_adjusted_		
Group 1 — ostomy	12.18 [10.40; 14.20]	10.67 [7.98; 13.05]	1.00*	0.0001^+^	0.0001^v^
Group 2 — obstructive resection	15.19 [12.17; 19.9]	16.39 [12.37; 18.10]	1.00*	0.618^+^	0.041^v^
Group 3 — bypass	17.69 [16.08; 18.43]	15.11 [13.28; 16.85]	1.00*	1.00^+^	0.133^v^
Norm — before ischemia	18.90 [17.98; 19.73]	18.74 [17.46; 19.90]	—	—	—

^*^ The statistical significance of the differences in the L index values between the distal and proximal parts of the intestine; ^+^ in the proximal part of the intestine in groups 1–3 compared with the norm; ^v^ in the distal section of the intestine in groups 1–3 compared with the norm.

## Discussion

The qualitative and quantitative changes in microcirculation detected in this study demonstrate the severity of circulatory disorders in the intestinal wall and contribute to our understanding of the post-ischemic damage and its pathogenesis. A decisive role in obtaining the *in vivo* data was played by the MM OCT technology, the informative value of which has been repeatedly confirmed by previous studies in the field of surgical gastroenterology [31–33, 38–40].

In the present study, we demonstrated for the first time that following enterostomy (group 1), a significant decrease (by 35–45% from the initial level) in the total length of perfused blood vessels occurred in both the proximal and distal portions of the intestine. In our view, these unidirectional changes indicate the simultaneous occurrence of two pathogenetic pathways in the wall of the stoma: ischemia and a decrease in the blood vessel tone. These data together with the histological results suggest that ostomy following post-ischemic resection is the least acceptable way of treatment: it is associated with the most pronounced destruction in the intestinal wall. This rapid deterioration and tissue damage in the stomatized intestine apparently necessitates excising of the stoma before an anastomosis is applied.

With a different technique (group 2), after keeping the blind ends of the intestine inside the abdominal cavity, there was some decrease in the total length of the perfused vessels as revealed by the OCA images. In this model (in contrast to that with a stoma), the overall tone of intramural vessels in the blind sections of the intestine showed no critical changes, which indicated the preservation of blood flow and its regulation. However, this technique of obstructive bowel resection was associated with unparalleled changes in the proximal and distal sections. According to the OCT and morphological data, in the proximal section, there were pronounced clinical manifestations of intra-intestinal hypertension, as well as histological signs of wall edema, and microthrombi in the intramural vessels. In the distal section, ischemia manifested in a statistically significant (p_adjusted_=0.041) decrease in the length of perfused intramural vessels as compared with the normal value. Despite the preserved viability of the intestinal wall in the interoperative period, such an imbalance can complicate healing after an anastomosis is imposed at the second stage of the treatment.

The least pronounced changes in microcirculation and microstructure were found in the group of animals with bowel bypass surgery (group 3); these relatively mild changes were presumably due to the continued intestinal passage and the maintenance of the uniform intra-intestinal pressure in the proximal and distal parts of the intestine. Changes in the lengths of intramural vessels ranged from 6 to 20% of the baseline but they were not statistically significant (p_adjusted_=1.00 in the proximal and p_adjusted_=0.133 in the distal section). It is important to note that despite the physiological and pathological evidence in favor of bowel shunting as the best surgical option preceding a delayed anastomosis, the reliable technique for such shunting operation is yet to be developed.

## Conclusion

Based on the macro- and microscopic data (appearance of the intestinal wall, its microcirculation and microstructure according to MM OCT, and histological examination) we find that the type of surgical treatment for the intestine after its emergency resection following acute mesenteric ischemia is crucial for the tissue viability in the period preceding the delayed anastomosis stage. Intestinal ostomy is accompanied by ischemic events and a decrease in the tone of blood vessels, pronounced edema and partial destruction of the serous membrane mucosa and mesothelium. Obstructive bowel resection followed by keeping the remnants in the abdominal cavity is associated with milder disturbances of microcirculation; however, it is associated with circulatory disorders unevenly expressed in the proximal and distal parts of the intestine, as well as with abnormalities of the intestinal wall microstructure (pronounced edema of the serous membrane and partial destruction of the villous epithelium). As a result, after 2 days, disproportionate changes occur in the bowel sections that remained after obstructive resection, which is seen as a poor prognostic factor for subsequent healing. The least pronounced and most balanced changes occur in the proximal and distal sections of the intestine after a bypass operation. However, a widespread use of this technique requires the development of reliable, safe, and effective bypass instruments.
